# Negative Stress Test Is Not Always Negative: Revisiting the Clinical Implications of Balanced Ischemia

**DOI:** 10.7759/cureus.30285

**Published:** 2022-10-14

**Authors:** Maddie Perdoncin, Ebubechukwu Ezeh, Utsab R Panta, Jason Mader

**Affiliations:** 1 Internal Medicine, Marshall University Joan C. Edwards School of Medicine, Huntington, USA; 2 Cardiology, Marshall University Joan C. Edwards School of Medicine, Huntington, USA

**Keywords:** coronary artery bypass grafting (cabg), coronary artery disease, severe triple vessel coronary artery disease, multivessel coronary artery disease (mvcad), cardio vascular disease

## Abstract

Myocardial perfusion imaging with technetium (99mTc) sestamibi often aids in the diagnosis of coronary artery disease. This diagnostic tool is highly important in dictating future clinical decision-making and determining whether patients may benefit from revascularization. However, it is not completely sensitive and may be unreliable in diagnosing coronary artery disease in patients with balanced perfusion deficits. Here, we present the case of a 45-year-old male with severe coronary artery disease and false-negative myocardial perfusion imaging.

## Introduction

Non-invasive cardiac imaging such as myocardial perfusion imaging (MPI) with technetium (99mTc) sestamibi allows for early diagnosis and evaluation of suspected coronary artery disease (CAD). They are also used for risk stratification in patients with established CAD [1]. This evaluation depends on differential blood flow through the coronary arteries. However, some patients will demonstrate adequate perfusion of myocardial cells irrespective of CAD. This leads to a false negative myocardial perfusion scintigram [2]. This unusual presentation results in numerous missed diagnoses of high-risk CAD and ultimately leads to poor cardiovascular outcomes.

## Case presentation

**Figure 1:**
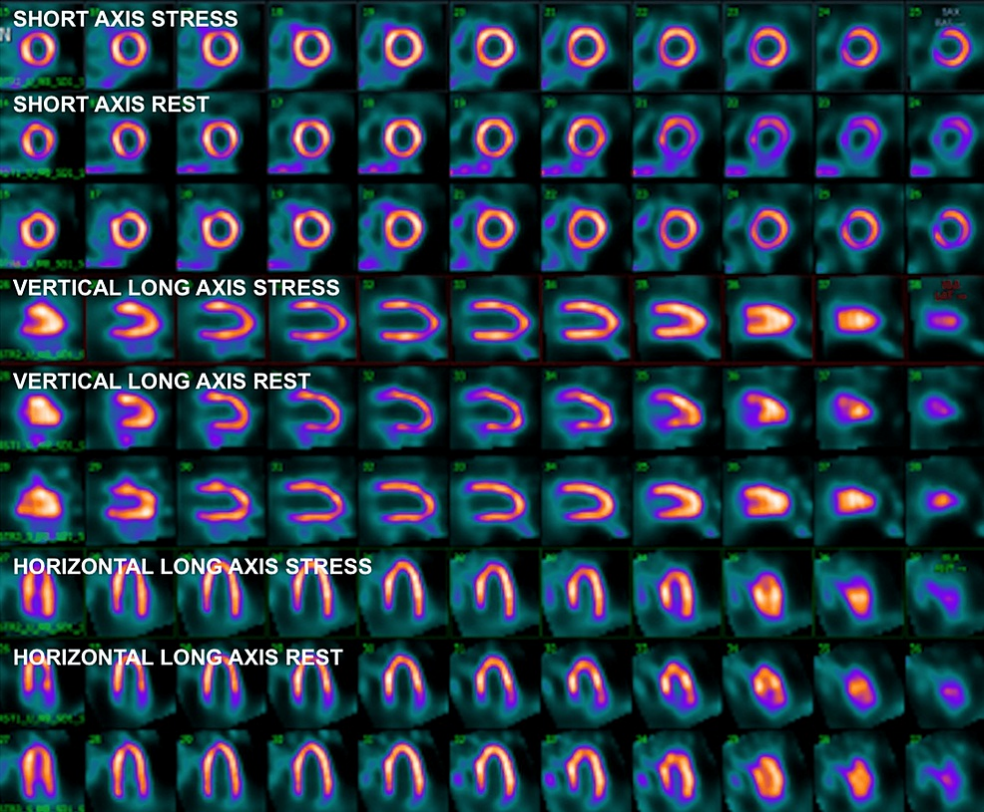
Myocardial perfusion imaging demonstrating normal stress test.

**Figure 2:**
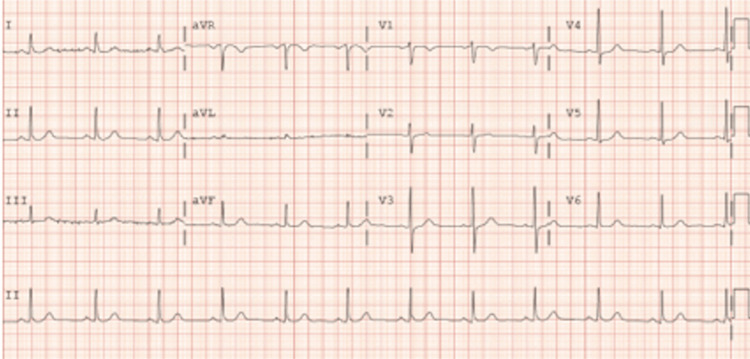
Demonstrating unremarkable EKG.

**Figure 3:**
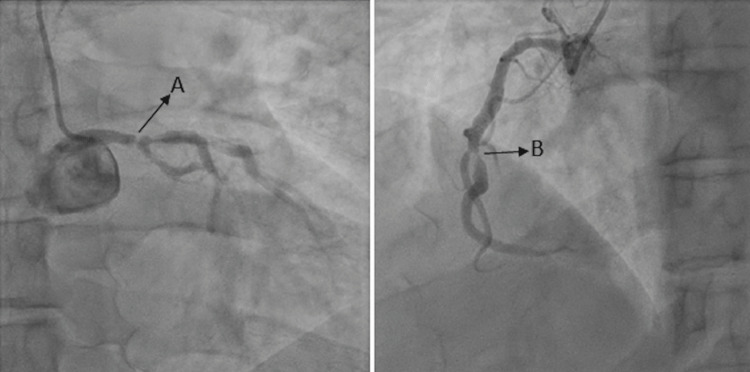
Left heart catheterization showing stenosis of the distal left main coronary artery (A) and mid-right coronary artery (B).

A 45-year-old male with a significant past medical history of mixed hyperlipidemia and hypertension presented with a one-month history of substernal exertional chest pain that radiated to both upper extremities and was relieved by rest. Notably, the patient presented with similar symptoms two weeks prior. Then, troponins were negative, and he had a normal stress test (Figure 1). On this presentation, vital signs were normal, and physical exam findings were unremarkable. The troponin trends recorded were 62, 83, and 88 (reference range 3.0-58.0) picogram/millimeter. The electrocardiogram showed no acute ischemic changes (Figure 2). Due to ongoing chest pain, the decision was made to perform left heart catheterization (LHC). The LHC showed 90-95% stenosis in the distal left main coronary artery, 70-80% stenosis in the second diagonal artery, and 99% stenosis in the mid-right coronary artery (Figure 3). The cardiothoracic team was consulted and a coronary artery bypass grafting was done followed by the initiation of guideline-directed medical therapy. His symptoms improved and he was discharged to cardiac rehabilitation.

## Discussion

99mTc sestamibi evaluates blood flow through the heart by distributing it into tissues with high mitochondrial content. Those tissues with high metabolic activity have increased uptake of the tracer, while tissues that have undergone damage, as in the case of myocardial infarction, will show decreased uptake of the tracer [3]. The photons emitted by the tracer are detected by a gamma camera, which allows the tracer to be visualized [4]. Although this is typically a reliable method to diagnose CAD, there are some instances in which a false negative may result, as was the case with our patient.

A study by Nakanishi et al. investigated the frequency of false negative results on myocardial perfusion scanning due to balanced ischemia. They found that out of 580 patients with normal MPI results, 7.2% had high-risk triple vessel disease as determined by invasive coronary angiography [5]. 

Balanced ischemia is one cause of a false-negative MPI result. This phenomenon occurs because MPI relies on the vessel that receives the highest level of perfusion as the standard to compare the remaining vessels in the heart. In severe triple-vessel CAD, the vessel with the highest flow already has a minimal level of perfusion; therefore, the remainder of the vessels will be analyzed as receiving normal levels of blood flow. This results in a false-negative scan [4]. Other reasons for a false-negative scan include caffeine ingestion, which results in decreased coronary vasodilation, as well as plateauing of radio-tracer at high rates of blood flow [5].

There are some diagnostic alternatives that may be considered for MPI. This patient may have been a good candidate for cardiac CT with coronary angiography as an initial modality to look for CAD. A stress echocardiogram is another option; however, this method is time-consuming and often results in poor-quality images [5]. Magnetic resonance imaging has also been proposed but is known to have the limitation of not being able to detect perfusion abnormalities in over 40% of patients with multi-vessel CAD [5]. Because these alternatives to MPI are not ideal, it is important to consider some predictive factors that can be used to approximate disease risk in patients with a normal MPI. The most important risk factors were pretest probability of CAD >66%, mild perfusion defects that were too small to be considered an abnormality, a high transient ischemic dilatation (TID) of the left ventricle, and abnormal stress and rest ejection fraction response. Thoughtful consideration of these high-risk factors may improve the detection of CAD in patients with normal MPI [5]. For example, patients with a pretest probability for CAD >66% will benefit from invasive evaluation of the coronary arteries since a negative MPI does not rule out CAD in such patients.

## Conclusions

Multi-vessel CAD has been reported as one of the causes of false-negative myocardial perfusion scans. Our patient had severe three-vessel CAD and a false-negative MPI. Although MPI is reported as one of the most sensitive non-invasive tests for CAD, care should be taken in interpreting the results in high-risk patients. Persistent symptoms in such high-risk patients may warrant further diagnostic assessment for CAD.
